# Preventing Ventilator-Associated Lung Injury: A Perioperative Perspective

**DOI:** 10.3389/fmed.2016.00025

**Published:** 2016-05-30

**Authors:** Satoshi Kimura, Nicoleta Stoicea, Byron Rafael Rosero Britton, Muhammad Shabsigh, Aly Branstiter, David L. Stahl

**Affiliations:** ^1^Department of Anesthesiology, The Ohio State University Wexner Medical Center, Columbus, OH, USA

**Keywords:** lung protective ventilation, low tidal volume ventilation, ventilator-induced lung injury, ventilator-associated lung injury, acute respiratory distress syndrome, general anesthesia

## Abstract

**Introduction:**

Research into the prevention of ventilator-associated lung injury (VALI) in patients with acute respiratory distress syndrome (ARDS) in the intensive care unit (ICU) has resulted in the development of a number of lung protective strategies, which have become commonplace in the treatment of critically ill patients. An increasing number of studies have applied lung protective ventilation in the operating room to otherwise healthy individuals. We review the history of lung protective strategies in patients with acute respiratory failure and explore their use in patients undergoing mechanical ventilation during general anesthesia. We aim to provide context for a discussion of the benefits and drawbacks of lung protective ventilation, as well as to inform future areas of research.

**Methods:**

We completed a database search and reviewed articles investigating lung protective ventilation in both the ICU and in patients receiving general anesthesia through May 2015.

**Results:**

Lung protective ventilation was associated with improved outcomes in patients with acute respiratory failure in the ICU. Clinical evidence is less clear regarding lung protective ventilation for patients undergoing surgery.

**Conclusion:**

Lung protective ventilation strategies, including low tidal volume ventilation and moderate positive end-expiratory pressure, are well established therapies to minimize lung injury in critically ill patients with and without lung disease, and may provide benefit to patients undergoing general anesthesia.

## Introduction

Atelectasis is a common adverse effect of general anesthesia associated with both hypoxemia and postoperative respiratory complications (1–3). Mechanical ventilation (MV) may be used to combat atelectasis by prescribing relatively large tidal volumes (V_T_) [up to 15 mL/kg predicted body weight (PBW)] (4, 5). However, we now know that using high V_T_ ventilation in the intensive care unit (ICU) increases mortality (6), and that MV itself can cause lung injury, through a process referred to as ventilator-induced lung injury (VILI) (7). VILI occurs through a number of different mechanisms.

Barotrauma, defined as pulmonary injury from elevated transpulmonary pressures, was the first widely recognized element of VILI (7, 8). Since it was subsequently demonstrated that humans could generate high airway pressures without causing lung injury, clinical focus shifted to volutrauma, hyperaeration caused by high V_T_ leading to lung injury (7, 8). Conversely, atelectrauma occurs when tidal ventilation at low airway pressures causes cyclic opening and collapse of unstable lung units (7, 9). This repeated cycling generates disruptive forces on the alveolar basement membranes and augments lung injury (7). Since the early 1990s, numerous studies have shown that MV can trigger the release of pro-inflammatory cytokines and the recruitment of neutrophils (10–13). This biological reaction in response to mechanical forces is known as biotrauma (8, 9, 14, 15).

Clinically, it can be difficult to delineate MV as the precise cause of lung injury. Therefore, when lung injury is concurrent to, but not necessarily caused by, MV, the term ventilator-associated lung injury (VALI) is used (7). The potentially detrimental effects of MV were recognized as early as 1960s (16) when canine studies demonstrated that lung overdistension resulted in an absence of surfactant and subsequent atelectasis (17). Attempts to prevent lung overdistention by therapeutically reducing V_T_ in humans in the ICU became feasible with acceptance of permissive hypercapnia (18).

Over the past 15 years, studies of patients with acute respiratory failure have resulted in the development of a number of lung protective strategies applied to minimize VALI. These strategies include low V_T_ to limit volutrauma, higher positive end-expiratory pressure (PEEP) to prevent atelectrauma, and recruitment maneuvers (i.e., application of temporary high airway pressures to reinflate collapsed lung units) (19). The successful application of these strategies in acute respiratory failure has led to increased interest in applying these principles to other patient populations. What remains controversial is whether the principles of lung protective ventilation derived from ICU patients with acute respiratory failure have applicability to otherwise healthy patients in the operating room.

## Materials and Methods

A systematic literature search was performed to identify clinical studies of lung protective ventilation strategies. We searched PubMed, EMBASE, and the Cochrane Library from inception to May 2015 with restriction to human studies published in English using a combination of standardized search terms and keywords to cover the topics of acute lung injury, acute respiratory distress syndrome (ARDS), MV, lung protective ventilation, low V_T_ ventilation, and general anesthesia. We reviewed articles that investigated the effects of lung protective ventilation strategies. We included randomized controlled trials (RCT), cohort, cross-sectional, and before-and-after studies. References of all studies were inspected for additional articles that were not identified by the electronic database search. We removed non-human studies, case reports, abstracts, and any other study where the full text was unavailable. Additionally, we excluded studies that specifically examined one-lung ventilation.

## Results

### Origin of Lung Protective Strategies: Patients with ARDS and Acute Respiratory Failure in the ICU

In 1998, Amato et al. described a lung protective strategy, which included a V_T_ of <6 mL/kg, PEEP above the lower inflection point, and permissive hypercapnia, was associated with improved survival at 28 days, an improved rate of weaning from MV, and a lower rate of barotrauma in patients with ARDS (20). In 2000, The Acute Respiratory Distress Syndrome Network reported that lower V_T_ (6 mL/kg of PBW) resulted in decreased mortality and increased ventilator-free days compared with V_T_ of 12 mL/kg of PBW (6). An increase of 1 mL/kg PBW in initial V_T_ has been associated with a 23% increase in ICU mortality risk (21). In patients without ARDS, a retrospective cohort study demonstrated the use of large V_T_ was the main risk factor associated with the development of lung injury (odds ratio 1.3 for each milliliter/kilogram above 6 mL/kg PBW, *p* < 0.001) (22). And a prospective study conducted by the same group concluded that initial ventilator settings of high V_T_ (odds ratio 2.6 for V_T_ > 700 mL) and high peak airway pressure (odds ratio 1.6 for peak airway pressure >30 cmH_2_O) were associated with the development of ARDS in patients without ARDS at the onset of MV (23).

### Lung Protective Ventilation during General Anesthesia in Operating Room

Although lung protective ventilation strategies are widely used in the ICU, it remains unclear if these strategies can be directly translated into use in the operating room. Respiratory complications are low in patients undergoing general anesthesia, unlike the ICU (24). Therefore, it may take a much larger number of patients to improve outcomes using lung protective strategies, and there are risks associated with lung protective ventilation, including increased alveolar collapse (25), increased right ventricular afterload (26), hypercapnia and respiratory acidosis (27), and worsened metabolic acidosis (27). Studies on intraoperative MV can be divided into those that target surrogate markers of lung inflammation/injury, clinical outcomes, or systemic reviews and meta-analyses. We examine these studies to better understand the risks and benefits of lung protective ventilation in the operating room (Table 1).

**Table 1:** **Randomized controlled trials comparing lung protective strategies with low tidal volumes to conservative ventilation in operating room**.

Type of surgery	Source	Patients’ population	Lung protective ventilation (V_T_)	Conservative ventilation (V_T_)	Primary outcome
Cardiac surgery	Chaney et al.(36)	Adults patients undergoing elective on-pump CABG surgery (*n* = 25)	6 mL/kg with 5 cmH_2_O of PEEP (*n* = 12)	12 mL/kg with 5 cmH_2_O of PEEP (*n* = 13)	Pulmonary mechanics
	Koner et al. (34)	Adult patients undergoing elective on-pump CABG grafting surgery (*n* = 44)	6 mL/kg with 5 cmH_2_O of PEEP (*n* = 15)	10 mL/kg with 5 cmH_2_O of PEEP (*n* = 14) or zero PEEP (*n* = 15)	TNF-α and IL-6 levels
	Zupancich et al. (28)	Adult patients undergoing elective on-pump CABG surgery (*n* = 40)	8 mL/kg with 10 cmH_2_O of PEEP (*n* = 20)	10–12 mL/kg with 2–3 cmH_2_O of PEEP (*n* = 20)	IL-6 and IL-8 in BAL fluid and plasma
	Reis Miranda et al. (29)	Adult patients undergoing elective on-pump CABG or valve surgery (*n* = 62)	4–6 mL/kg with 10 cmH_2_O of PEEP during (*n* = 22) or after (*n* = 18) surgery	6–8 mL/kg (*n* = 22) with 5 cmH_2_O of PEEP	IL-6, IL-8, IL-10, TNF-α, and interferon-γ
	Sundar et al.(43)	Adult patients undergoing elective cardiac surgery (*n* = 149)	6 mL/kg with PEEP according to ARDS Network investigators (6) (*n* = 75)	10 mL/kg with PEEP according to ARDS Network investigators (6) (*n* = 74)	Time to extubation

Abdominal surgery	Wrigge et al. (31)	Adult patients undergoing major abdominal surgery (*n* = 30)	6 mL/kg with 10 cmH_2_O of PEEP (*n* = 15)	12 or 15 mL/kg with zero PEEP (*n* = 15)	TNF, IL-1, IL-6, IL-8, IL-10, and IL-12
	Weingarten et al. (37)	Adult patients aged >65 years undergoing major open abdominal surgery under general anesthesia (*n* = 40)	6 mL/kg with 12 cmH_2_O of PEEP and recruitment maneuvers (*n* = 20)	10 mL/kg with no PEEP and no recruitment maneuvers (*n* = 20)	Oxygenation, pulmonary mechanics, hemodynamic stability
	Treschan et al. (40)	Adult patients undergoing elective upper abdominal surgery lasting ≥3 h under combined general and epidural anesthesia (*n* = 101)	6 mL/kg with 5 cmH_2_O of PEEP (*n* = 50)	12 mL/kg with 5 cmH_2_O of PEEP (*n* = 51)	Lung function
	Severgnini et al. (38)	Adult patients undergoing elective open abdominal surgery ≥2 h (*n* = 55)	7 mL/kg with 10 cmH_2_O of PEEP and recruitment maneuvers (*n* = 28)	9 mL/kg with zero PEEP (*n* = 27)	Modified Clinical Pulmonary Infection Score, gas exchange, and pulmonary functional tests
	Futier et al. (5)	Adults patients at intermediate to high risk of pulmonary complications undergoing major abdominal surgery (*n* = 400)	6–8 mL/kg with 6–8 cmH_2_O of PEEP and recruitment maneuvers (*n* = 200)	10–12 mL/kg with no PEEP and no recruitment maneuvers (*n* = 200)	A composite of major pulmonary and extrapulmonary complications occurring by day 7 after surgery

Others	Cai et al. (39)	Adults patients aged 20–50 years with body mass index undergoing elective excision of intracranial lesions (*n* = 16)	6 mL/kg without PEEP (*n* = 8)	10 mL/kg without PEEP (*n* = 8)	Atelectasis (by CT and ABG)
	Memtsoudis et al. (32)	Adult patients undergoing elective lumbar decompression and fusion in prone position under general anesthesia (*n* = 26)	6 mL/kg with 8 cmH_2_O of PEEP (*n* = 13)	12 mL/kg with zero PEEP (*n* = 13)	Plasma levels of IL-6 and IL-8, and urinary levels of desmosine
	Choi et al. (33)	Adult patients undergoing a surgical procedure ≥5 h (*n* = 40)	6 mL/kg with 10 cmH_2_O of PEEP (*n* = 21)	12 mL/kg without PEEP (*n* = 19)	Markers of coagulation and fibrinolysis
	Wolthuis et al. (30)	Adult patients undergoing a surgical procedure ≥5 h (*n* = 40)	6 mL/kg with 10 cmH_2_O of PEEP (*n* = 21)	12 mL/kg without PEEP (*n* = 19)	Polymorphonuclear cell influx, changes in levels of inflammatory markers, and nucleosomes in BAL fluid and/or blood
	Determann et al. (35)	Adult patients undergoing a surgical procedure ≥5 h (*n* = 40)	6 mL/kg with 10 cmH_2_O of PEEP (*n* = 21)	12 mL/kg without PEEP (*n* = 19)	Local and systemic levels of Clara cell protein

*V_T_, tidal volumes; CABG, coronary artery bypass grafting; PEEP, positive end-expiratory pressure; TNF, tumor necrosis factor; IL, interleukin; BAL, bronchoalveolar lavage; CPB, cardiopulmonary bypass; ARDS, acute respiratory distress syndrome; CT, computed tomography; ABG, arterial blood gas*.

#### Lung Protective Ventilation, Pulmonary Biomarkers, and Lung Mechanics

Inflammatory biomarkers can be used as surrogate outcomes for lung inflammation or injury in patients receiving MV. For example, one RCT compared 40 patients undergoing elective coronary artery bypass grafting (CABG) receiving high V_T_/low PEEP or low V_T_/high PEEP (28). After 6 h of MV, interleukin (IL)-6 and IL-8 levels were elevated in bronchoalveolar lavage (BAL) fluid and plasma in patients who received higher V_T_/low PEEP, suggesting alveolar lung inflammation (28). The attenuation of IL-8, but not IL-6 levels by lung protective ventilation has been replicated in other surgical patients undergoing cardiopulmonary bypass (29) as well as elective surgery (30) but not in major abdominal, thoracic (31), or spinal surgery (32). IL-10 has a similarly mixed profile in response to lung protective MV (29). High V_T_/zero end-expiratory pressure (ZEEP) MV does appear to lead to procoagulant activation in the alveolar space (33). Many other inflammatory markers, including tumor necrosis factor-alpha (TNF-α), interferon (IFN)-γ, IL-1, IL-1α, IL-1β, IL-6, IL-12, macrophage inflammatory protein 1α, and macrophage inflammatory protein 1β, or other markers of lung epithelial injury have not been demonstrated to change in response to lung protective ventilation (29–31, 34, 35).

Other studies have demonstrated improved perioperative pulmonary mechanics as a surrogate outcome. One single-center RCT included 25 patients undergoing elective CABG showed a greater reduction in both dynamic and static lung compliance in patients receiving conventional MV, suggesting lung protective MV resulted in improved pulmonary mechanics (36). RCTs in abdominal surgery had varied results showing improved (37), unchanged (38, 39), and degraded (40) pulmonary mechanics in patients receiving lung protective MV. An improvement in the partial pressure of arterial oxygen (PaO_2_)/Fraction of inspired oxygen (FiO_2_) ratio in abdominal surgery patients appears to reflect the use of recruitment maneuvers more than any particular lung protective strategy (37, 40).

#### Lung Protective Ventilation and Clinical Outcomes

Several retrospective studies have investigated the association between MV strategies in the operating room and clinical outcomes. A retrospective evaluation of patients admitted to the ICU with postoperative hypoxemic respiratory failure, requiring MV, showed that a high V_T_ to ideal body weight (IBW) ratio was an independent risk factor for the development of ARDS (41). While a retrospective study of billing data for over 69,000 patients undergoing non-cardiac surgery did not show any effect of V_T_ on postoperative respiratory complications (24). Another retrospective study of more than 29,000 patients reported that V_T_ of 6–8 mL/kg IBW were associated with a significant increase in 30-day mortality compared with V_T_ of 8–10 mL/kg IBW (hazard ratio 1.6, *p* = 0.0002) (42). The authors hypothesized that the increased mortality may be related to the combination of lower V_T_ and low PEEP (≤5 cmH_2_O) (42).

Prospective RCTs of MV in the operating room have specifically targeted high-risk surgical groups, primarily cardiac and abdominal surgery patients. One single-center RCT investigated duration of intubation for cardiac surgery patients by comparing ventilation with low vs. high V_T_ ventilation. A higher proportion of patients were extubated 6 h postoperatively in the low V_T_ group (37.3 vs. 20.3%; *p* = 0.02) and fewer patients in the low V_T_ group required re-intubation (0.3 vs. 9.5%; *p* = 0.03) (43). In two RCTs of patients undergoing open abdominal surgery, lower V_T_ ventilation combined with PEEP and recruitment maneuvers decreased respiratory complications as measured by two different postoperative pulmonary complication scores when compared to higher V_T_ ventilation with ZEEP and no recruitment maneuvers (5, 38). The IMPROVE trial also found that patients receiving lung protective ventilation and recruitment maneuvers had decreased postoperative non-invasive ventilation and intubation requirements (5.0 vs. 17.0%, *p* = 0.001), and shorter length of hospital stay (mean difference, −2.45 days, *p* = 0.006) (5). However, in 101 patients undergoing upper abdominal surgery who were randomized to low V_T_ ventilation (6 mL/kg PBW) with 5 cmH_2_O PEEP or to higher V_T_ ventilation (12 mL/kg PBW) also with 5 cmH_2_O PEEP, there was no significant difference in postoperative pulmonary complications (40). In the largest prospective multicenter RCT, conducted by the Protective Ventilation (PROVE) Network Investigators, 900 patients were allocated either to a high level of PEEP (12 cmH_2_O) with recruitment maneuvers (higher PEEP group) or a low level of PEEP (≤2 cmH_2_O) without recruitment maneuvers (lower PEEP group) while receiving 8 mL/kg V_T_ (PROVHILO trial). In this study, postoperative pulmonary and extrapulmonary complications were not significantly different between the two groups although hypotension happened more frequently in the higher PEEP group (relative risk 1.29; *p* = 0.0016) and those in the higher PEEP group had a greater need for vasopressors than those in the lower PEEP group (relative risk 1.20; *p* = 0.0016) (44). The results of these trials suggest an important interaction between V_T_ and PEEP affecting clinical outcomes.

#### Lung Protective Ventilation Meta-Analyses

The recently updated Cochrane meta-analysis evaluated 12 studies with 1012 participants comparing specifically low vs. high V_T_ ventilation in the operating room (45). They found no difference in 30-day mortality (risk ratio (RR) 0.79, 95% confidence interval (CI) 0.40-1.54), but the low V_T_ cohort did show a decrease in postoperative pneumonia and a decreased requirement for both invasive and non-invasive postoperative ventilation (45). Decreased rates of pulmonary infection have been confirmed by other meta-analyses of lung protective ventilation (including interventions in addition to low V_T_) (46–48). These additional meta-analyses also failed to demonstrate a mortality benefit to intraoperative lung protective ventilation (46, 47).

#### Lung Protective Ventilation in Anesthetic Practice

While the data to support lung protective ventilation in the operating room remains unclear, it is worthwhile to examine the current state of anesthetic practice regarding MV. One cross-sectional analysis of intraoperative ventilation during elective abdominal surgery in the United States showed that ventilator settings were non-uniform and ventilation with V_T_ > 10 mL/ kg PBW is still common (17.5%). BMI ≥30, female gender, and height <165 cm may predispose certain patients to receive large V_T_ during general anesthesia as providers likely overestimate PBW in this patient group (49). Despite the high prevalence of high V_T_ ventilation in the operating room, a targeted study of the management of hypoxemic patients (defined by P/F ratio) in the operating room found V_T_, and peak inspiratory pressure (PIP) have been decreasing annually while PEEP has been increasing (50). Unfortunately, worsened hypoxemia (lower P/F ratio) correlated most closely with higher FiO_2_ and PIP, suggesting that patients who would most benefit from lung protective ventilation are not receiving it (50). Investigators in Australia performed an audit in 272 patients undergoing general anesthesia for three consecutive days in 2013. They showed that a median V_T_ was 9.5 mL/ kg PBW, suggesting that the practice of MV in the operating room does not always reflect recent studies (51). There are several ongoing studies investigating variation of MV setting within/between centers (52)^1^.

## Discussion

The translation of research from the ICU to the operating room can be difficult for several reasons. Patients receiving MV in the operating room during general anesthesia commonly lack the derangements in gas exchange and pulmonary mechanics seen in the ICU. While general anesthesia does induce atelectasis, the atelectasis is well-tolerated and relatively short-lived (1–3). Rates of respiratory complications remain low in most populations and the short duration of MV likely affects lung compliance less than a prolonged course of ventilation in ICU. Furthermore, ICU patients are more likely to have comorbid illnesses, such as cardiovascular instability and shock, acid–base abnormalities, and pro-inflammatory states, such as sepsis, which affect the goals of MV.

Based on published ICU data, studies of lung protective ventilation in the operating room generally have used 6–8 mL/kg PBW as low V_T_ ventilation (Table 1), which may not be the ideal V_T_ for surgical patients (42). Furthermore, studies in the perioperative setting have used low V_T_ combined with PEEP and/or recruitment maneuvers as the lung protective strategies. Although low V_T_ ventilation is clearly an important factor of the lung protective ventilation (53), it is still unclear how we should combine these “lung protective strategies,” including PEEP and recruitment maneuvers, into a unified best practice for MV in the operating room (44). A subsequent ongoing international multicenter RCT, aims to compare a ventilation strategy using higher levels of PEEP with recruitment maneuvers to one using lower levels of PEEP without recruitment maneuvers in obese patients at risk of postoperative pulmonary complications^2^. This study design hints at the holy grail for intraoperative ventilation: targeted interventions for specific patients. While still heterogeneous themselves, patients with acute respiratory failure and ARDS in the ICU are substantially more homogeneous than the morass of patients undergoing general anesthesia in the operating room. This heterogeneity may, in part, explain the disparate findings in previous studies. However, as we gain a better understanding of the risks and benefits of the various aspects of lung protective strategies, we can more effectively target-specific patient populations for intervention.

## Conclusion

As lung protective strategies, including low V_T_ ventilation, higher PEEP, and/or recruitment maneuvers, continue to evolve, they may also provide benefit to patients undergoing general anesthesia. Larger trials of specific ventilator strategies across different populations are required to evaluate different lung protective strategies and their interaction with different groups of surgical patients to clarify the benefit or harm of lung protective ventilation in the operating room. Furthermore, we need investigations into current practice strategies of clinical anesthesiologists, in order to help shape how new data are applied in the operating room.

## Author Contributions

SK made contributions to conception and design, participated in drafting the article, and revised it critically for important intellectual content. NS participated in drafting the article and revised it critically for important intellectual content. BB participated in drafting the article and revised it critically for important intellectual content. MS participated in drafting the article and revised it critically for important intellectual content. AB participated in drafting the article and revised it critically for important intellectual content. DS made contributions to conception and design, participated in drafting the article, and revised it critically for important intellectual content.

## Conflict of Interest Statement

The authors declare that the research was conducted in the absence of any commercial or financial relationships that could be construed as a potential conflict of interest.

## Footnotes

^1^IVENT. International Survery on VENTilation Practice (iVENT). In: PROVEnet [Internet]. Available from: https://sites.google.com/site/iventsurvey/.

^2^Technische Universität Dresden. Protective Ventilation with Higher Versus Lower PEEP during General Anesthesia for Surgery in Obese Patients (PROBESE). In: ClinicalTrials.gov [Internet]. Available from: http://clinicaltrials.gov/show/NCT02148692.
